# Different temporal weight-bearing tendencies of persons with right and left hemiplegia while sitting in a wheelchair

**DOI:** 10.1371/journal.pone.0262849

**Published:** 2022-01-27

**Authors:** Hunhee Kim, Taekyeong Lee, Kang Hee Cho, Gwang Moon Eom, Junghwa Hong

**Affiliations:** 1 Department of Control and Instrumentation Engineering, College of Science and Technology, Korea University, Sejong, Republic of Korea; 2 Department of Rehabilitation Medicine, School of Medicine, Chungnam National University, Chungnam, Republic of Korea; 3 School of Biomedical Engineering, Research Institute of Biomedical Engineering, Konkuk University, Choonju, Republic of Korea; 4 College of Medicine, Korea University, Seoul, Republic of Korea; University of Ottawa, CANADA

## Abstract

The tendency of persons with hemiplegia to sit for prolonged periods can cause excessive interface pressure (IP) on their buttocks. Due to the different neuromusculoskeletal conditions, different buttock IP relief methods are required for persons with left hemiplegia (LH) and right hemiplegia (RH). Therefore, this study investigates temporal characteristics of IP on the right and left buttocks for RH, LH, and able-bodied individuals (AB) sitting in a wheelchair for 30 min. Thirty-five males participated in the study: 13 LH, 12 RH, and 10 AB. In the initial adjustment phase, the participants maintained an erect sitting posture for 7 min (2 min for posture and 5 min for creep adjustments). After the adjustments, experiments were conducted for 30 min to measure the IP. In the experiments, significant right-sided asymmetries of the mean IP were found for each group (*P* < 0.05). The right buttocks of LH exhibited significantly more right-sided asymmetry of the mean IP than that of AB (*p* < 0.01). Moreover, the right buttocks of RH exhibited insignificant asymmetry of the mean IP compared to that of AB (*p* >0.21). The peak IPs of RH and LH were significantly higher than those of AB (*p* <0.05), and temporal changes of the mean and peak IP of hemiplegia were significant (*p* <0.05) and not significant (*p* >0.05), respectively. The RH exhibited affected-side weight-bearing based on the mean IP. In contrast, the LH relieved the mean IP on the affected-side buttock. Due to the right-sided asymmetric mean and high peak IP, hemiplegia in acute and recovery stages using wheelchairs can cause ulceration. Therefore, different rehabilitation approaches are required for the RH and LH to reduce the peak IP and avoid an uneven distribution of the mean IP.

## Introduction

Among the aging and aged societies, the number of persons with hemiplegia staying at home or in hospitals has dramatically increased. For example, in Korea, the number of persons with hemiplegia having permanent deficits in limb movement, gait, and trunk mobility is approximately 330,000 and is increasing by approximately 16,000 each year [1]. In the United States, strokes are the leading cause of motor disabilities in adults [2]. Therefore, persons with hemiplegia movement disorders have sedentary lifestyles and spend a significant amount of time in chairs or wheelchairs. The prolonged sitting tendencies of these persons can lead to serious health problems such as ulcerations on their buttocks.

The muscles on the hemiplegic side of stroke patients present neurological disorders that can cause asymmetrical body postures. A person with hemiplegia tends to bear heavier loads on their stronger side when they move [3–6]. Reference [7] suggested that avoiding loads on the weaker side causes unstable movement control and balance. In addition, the asymmetrical interface pressure (IP) distribution of hemiplegia is supported by significant unaffected-side loading asymmetry on the buttocks [8]. In contrast, one study postulated that the IP on the buttocks of a person with hemiplegia had asymmetrical distribution because they were supporting their weight with the affected side [9]. Moreover, no significant asymmetry of IP on the buttocks was found in the resting state when wheelchair propulsion tests were performed [10]. Therefore, studies considering the causes of asymmetrical buttock IP while sitting for persons with hemiplegia remain inconclusive.

The tendency of a person with hemiplegia to sit for long periods also causes gradual pelvic obliquity and tilting changes with respect to the amount of sitting time [8, 9]. The temporal changes in the pelvic angular motions of persons with hemiplegia while sitting may cause changes in the mean and peak IPs on the right and left buttocks and the asymmetrical buttock IP depending on the sitting time [8]. These temporal changes in buttock IP characteristics of persons with hemiplegia have not been studied and analyzed thus far. An investigation to understand the temporal changes in the IP characteristics can help prevent or treat and rehabilitate decubitus ulcers on the buttocks of persons with hemiplegia.

Note that the concentrations of IP on the buttocks are closely related to the peak IP, which is a leading cause of decubitus ulcers [11]. A study indicated that the higher IPs on the buttocks of the elderly compared to those of the able-bodied individuals (AB) increased the probability of ulcerations [12]. As the pathologies of the affected side could affect the unaffected-side buttock of persons with hemiplegia, some degree of atrophy of tissues may occur in the unaffected-side buttock, particularly for those in the acute and recovery stages. Therefore, a study is required to compare the IPs on each buttock side of persons with hemiplegia to those of AB. This could reveal whether IP on the unaffected-side buttock is affected by the atrophy and paralysis of the affected side of the hemiplegia. If the IPs on the unaffected-side buttock are greater due to the effects of the affected-side buttock, ulcerations can be developed even on the unaffected-side buttock of persons with hemiplegia. However, no related studies have been performed comparing the IPs on each buttock side.

The purpose of this study is to investigate that LH and RH would have different weight-releasing tendencies during sitting owing to different neuromusculoskeletal conditions. This study investigated the temporal changes in buttock IP characteristics for designated periods of sitting in a wheelchair recorded at 0, 5, 10, 15 20, 25, and 30 min for left hemiplegia (LH), right hemiplegia (RH), and AB. The asymmetries of the mean and peak IPs for the right and left buttocks at the designated recording times for each group were also studied. Additionally, differences in the mean IP, peak IP, and peak IP coordinates on the right and left buttocks in the transverse plane among three groups were examined separately for designated sitting times.

## Methods

### Participants

The study protocol was approved by the Chungnam National University College of Medicine (CNUH 2016-05-022). Written informed consent was obtained from each participant before the experiments. The patients were hospitalized and dependent on wheelchairs. All patients had a maximum of eight weeks post-stroke and no hemianopia. The criteria for participant recruitment were selected randomly in the condition, which were 1) left/right hemiplegia patients who a maximum of eight weeks post-stroke without no hemianopia and impossible to independent walking alone, 2) able-bodied subjects who have no disabilities, 3) All participants have no other musculoskeletal condition or any known cardiovascular, pulmonary, and neurological disorders (Table 1). The experiment was performed in motion analysis room at the Chungnam National University College of Medicine. The RH, LH, and AB sat with an erect posture before the experiments and then in a relaxed posture during the experiments to reveal the effects of gradual temporal changes in sitting posture on the characteristics of buttock IP.

**Table 1:** Participated subjects (LH: 13 males, RH: 12 males, and AB: 10 males).

No.	Sex	Age (year)	Height (cm)	Weight (kg)	Remarks	Mean ± SD	
1	male	46	162.6	69.8	LH	age: 48.08±2.40, height: 167.54±3.26, weight: 67.12±3.32	age: 47.60±2.43, height: 168.02±3.27, weight: 67.72±3.16
2	male	48	163.8	62.7	LH		
3	male	46	167.9	72.6	LH		
4	male	49	166.5	71.9	LH		
5	male	45	170.5	67.4	LH		
6	male	46	164.1	64.8	LH		
7	male	48	164.3	65.2	LH		
8	male	48	169.5	67.1	LH		
9	male	47	170.8	66.6	LH		
10	male	49	164.8	65.9	LH		
11	male	54	170.7	71.3	LH		
12	male	48	171.2	63.7	LH		
13	male	51	170.9	63.6	LH		
14	male	48	160.1	72.0	RH	age: 47.08±2.47, height: 168.58±3.33, weight: 68.37±2.99	
15	male	47	170.1	70.8	RH		
16	male	46	166.8	66.7	RH		
17	male	51	169.8	70.9	RH		
18	male	46	170.3	69.8	RH		
19	male	50	171.3	70.4	RH		
20	male	46	167.9	68.1	RH		
21	male	47	164.8	63.7	RH		
22	male	41	169.6	69.2	RH		
23	male	47	171.3	70.6	RH		
24	male	48	169.4	64.3	RH		
25	male	48	171.5	63.9	RH		
26	male	28	175.6	78.1	AB	age: 34.90±5.20, height: 174.09±3.30, weight: 74.89±3.28	
27	male	39	173.5	76.7	AB		
28	male	40	170.8	67.9	AB		
29	male	36	167.2	72.4	AB		
30	male	39	172.6	75.2	AB		
31	male	34	177.2	78.9	AB		
32	male	38	174.4	75.9	AB		
33	male	37	174.8	72.7	AB		
34	male	24	178.5	73.9	AB		
35	male	34	176.3	77.2	AB		

LH, RH and AB are the left hemiplegia, right hemiplegia, and able-bodied people, respectively.

### Apparatus

The IP sensor used in this study was the CONFORMat^TM^ pressure-mapping (Tekscan Inc, Boston, Massachusetts, USA), which consists of a flexible thin-film containing several arrays of sensing cells with an actual sensing area of 471 mm (anteroposterior) × 471 mm (mediolateral) and a total of 1,024 sensing cells. The calibration device and procedures provided by the manufacturer were used to calibrate the pressure measurement system. Moreover, pre-measurement was started 5 min before the actual measurement after the participant sat on the IP sensor to minimize errors due to creep. A basic manual wheelchair with a fabric seat was used to measure IP on the buttocks during sitting (Daese Wheelchair Co., Republic of Korea). Seat cushions were not used in the experiments to maximize the local effects of IPs on the bony prominences, muscles, and skin of the buttocks of each participant.

### Experimental procedure

The experiments were conducted using the following procedures. First, the brake of the test wheelchair was activated. Subsequently, the calibrated IP sensor was placed on the fabric seat of the wheelchair in the laboratory at an ambient temperature of 20 ˚C. The participants sat on the IP sensor located on the fabric seat and were asked not to place their upper limbs on the wheelchair armrests. Thus, the arms of the participants rested on their thighs during the experiments. The height of the wheelchair footrests was adjusted to ensure that the femurs of the participant were parallel to the horizontal seat base frame of the wheelchair during the experiments. Each participant was asked to maintain an erect sitting posture for 7 min (2 min for posture adjustment and subsequently 5 min for creep adjustment). After the adjustments, IP measurement was started. We also asked the participant to stay relaxed as much as possible during the experiment. The experiment lasted 30 min (excluding the 7 min for posture and creep adjustments). Data on buttock IPs were obtained at a sampling rate of 1/min for 30 min [13]. After the experiment, a rest period was set for 30 min for creep recovery of the IP sensors.

### Data analysis

**Fig 1:**
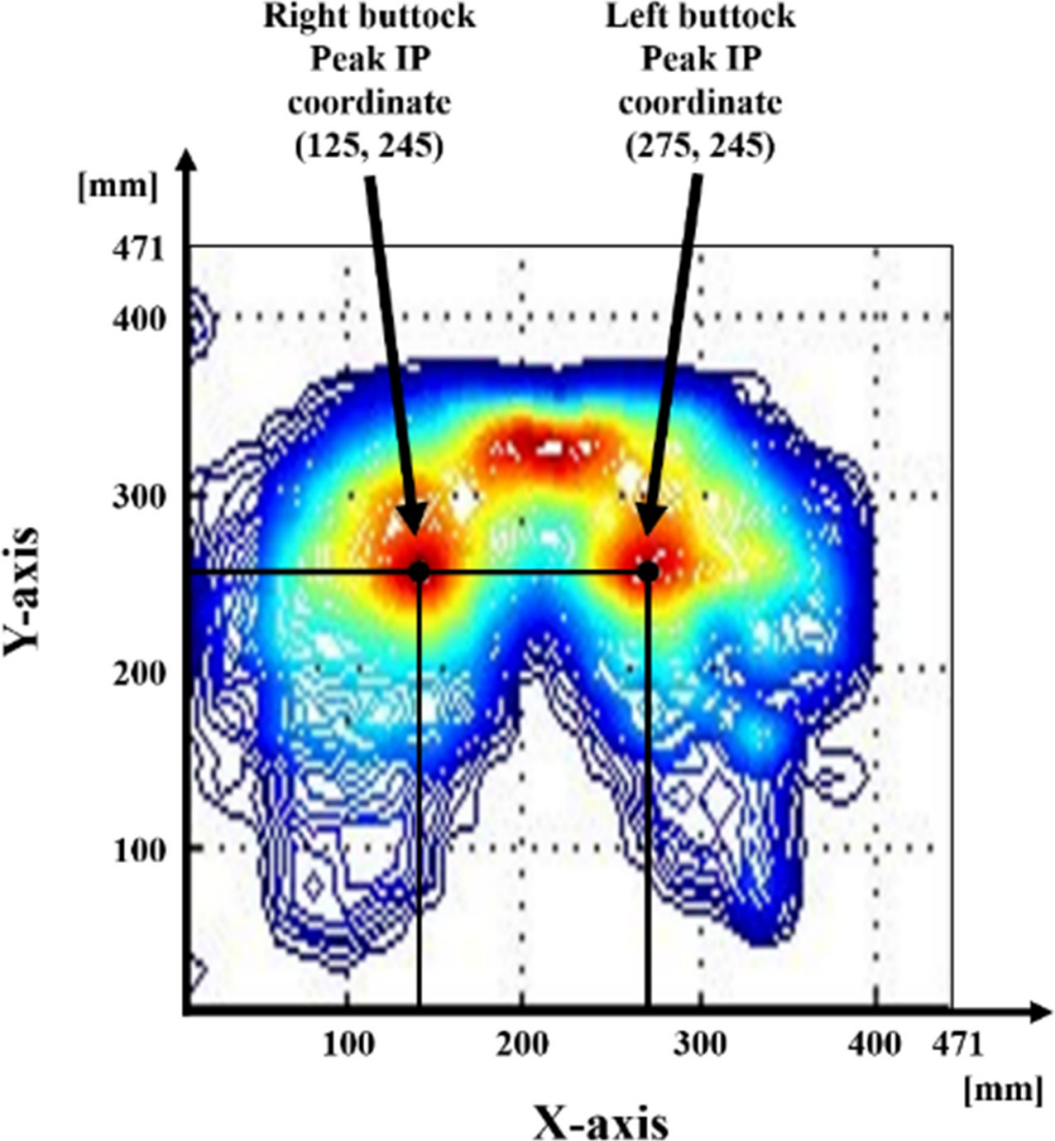
Example of the peak IP coordinates (in the case of AB at 0 min after sitting).

The data acquired from buttock IPs were separated according to the right and left buttocks. Consequently, the mean and peak IPs were obtained for each buttock. The measured mean and peak IPs for the right and left buttocks were classified for the RH, LH, and AB. Because the number of participants in each group does not represent a normal distribution, data analyses were performed using non-parametric analysis. All statistical analyses were performed using the SPSS software (version 23, SPSS Inc., Chicago, USA). To analyze the locations of peak IPs on the right and left buttocks, we used as the origin the most anterior right lateral point of the actual sensing area of the IP sensor in the transverse plane, as shown in Fig 1.

## Results

For the experiments, thirty-five males participated in this study: 13 with LH (age: 48.08 ± 2.40 years, height: 167.54 ± 3.26 cm, weight: 67.12 ± 3.32 kg), 12 with RH (age: 47.08 ± 2.47 years, height: 168.58 ± 3.33 cm, weight: 68.37 ± 2.99 kg), and 10 AB (age: 34.90 ± 5.20 years, height: 174.09 ± 3.30 cm, weight: 74.89 ± 3.28 kg) (Table 1). Then, the asymmetries of the mean and peak IPs and the temporal changes in IP characteristics was analyzed.

### Asymmetries of the mean and peak IPs

For this study, asymmetry is defined as the difference between the IPs on the right and left buttocks at a designated recording time for each group. As shown in Table 2, the asymmetries of the mean IPs for the RH, LH, and AB at all sitting times were statistically significant (*p* < 0.05). The RH, LH, and AB showed significant right-sided asymmetries of the mean IPs on the buttocks for all sitting times. In contrast, insignificant asymmetry of the peak IP for the RH, LH, and AB was found during sitting periods (p > 0.05). Although statistically insignificant, right-sided peak IPs on the buttocks of the RH, LH, and AB were higher than left-sided peak IPs for all recording times (Table 3).

**Table 2:** Related-samples Wilcoxon signed rank test results for determining asymmetries of the mean IPs at all recording times for the AB, RH and LH.

Mean IPs									
Time (min)	AB			RH			LH		
	R.Buttock (Mean ± SD)	L.Buttock (Mean ± SD)	p-value	R.Buttock (Mean ± SD)	L.Buttock (Mean ± SD)	p-value	R.Buttock (Mean ± SD)	L.Buttock (Mean ± SD)	p-value
0	26.51±3.16	22.36±2.18	0.007*	26.75±6.46	23.26±3.84	0.010*	27.10±2.03	21.09±3.03	0.001*
5	27.95±3.66	23.32±2.49	0.007*	27.96±7.03	23.99±4.12	0.005*	28.83±2.51	21.58±3.34	0.001*
10	28.64±3.79	23.53±2.52	0.005*	27.27±5.88	23.88±4.17	0.003*	28.54±2.63	22.11±3.19	0.001*
15	28.23±3.34	23.34±2.40	0.005*	28.27±5.96	25.10±4.84	0.002*	28.87±2.24	22.32±3.47	0.001*
20	28.57±4.15	23.43±3.48	0.005*	28.40±6.48	24.77±4.31	0.005*	28.54±2.36	21.48±3.90	0.001*
25	29.56±4.05	24.25±2.98	0.005*	29.18±6.86	24.62±4.92	0.003*	29.17±2.88	21.53±3.68	0.001*
30	29.93±3.59	24.60±3.04	0.005*	27.94±6.18	23.76±4.21	0.005*	28.94±2.12	21.12±3.48	0.001*

Wilcoxon signed ranks test.

R.buttock = Right buttock, L.buttock = Left buttock.

*p < 0.05, the mean IP on the right buttock significantly greater than the mean IP on left buttock.

The unit of the asymmetries is mmHg.

**Table 3:** Related-samples Wilcoxon signed rank test results for determining asymmetries of the peak Ips at all recording times for the AB, RH and LH.

Peak IP									
Time (min)	AB			RH			LH		
	R.b (Mean±SD)	L.b (Mean±SD)	p-value	R.b (Mean±SD)	L.b (Mean±SD)	p-value	R.b (Mean±SD)	L.b (Mean±SD)	p-value
0	194.48±45.29	183.65±35.34	0.139	244.07±8.91	226.94±27.01	0.099	230.06±26.64	219.15±20.50	0.158
5	196.00±40.76	184.55±35.36	0.093	247.85±5.74	226.84±25.51	0.050	233.67±24.82	221.24±23.46	0.279
10	198.79±39.82	187.27±33.07	0.139	248.28±5.92	228.17±27.28	0.060	241.91±14.88	232.25±25.52	0.345
15	196.28±46.97	185.10±34.85	0.169	249.39±5.52	228.83±24.42	0.050	243.46±17.37	229.25±20.75	0.075
20	198.52±47.60	185.29±28.32	0.169	246.06±7.29	227.66±26.41	0.060	244.58±20.28	228.76±17.05	0.055
25	200.65±44.64	185.68±28.10	0.093	246.54±8.81	226.61±25.60	0.065	239.61±19.35	230.75±20.01	0.173
30	201.50±43.64	189.18±27.36	0.241	244.23±9.71	224.60±26.28	0.060	235.10±19.81	220.55±20.51	0.055

Wilcoxon Signed Ranks Test.

R.b = Right buttock, L.b = Left buttock.

The unit of the asymmetries is mmHg.

Because RH, LH, and AB similarly showed significant right-sided asymmetries for the mean IPs, the difference of asymmetry (the mean IP of the right buttock and that of the left buttock) of the mean IP was analyzed. The difference of asymmetry compares the relative sidedness of the mean IPs for RH and LH to the mean IP for the AB. As shown in Table 4, the right buttock of LH showed significantly more right-sided asymmetry of the mean IP than that of the AB at all recording times (*p* <0.05). In contrast, the right buttock of RH showed insignificant asymmetry of the mean IP compared to that of the AB at all recording times (*p* >0.05).

**Table 4:** The difference of asymmetries of the mean IP for the AB, RH and LH.

The difference of asymmetries (Mean IPs)						
Time (min)	AB-RH			AB-LH		
	Diff.AB (Mean ± SD)	Diff.RH (Mean ± SD)	p-value	Diff.AB (Mean ± SD)	Diff.RH (Mean ± SD)	p-value
0	4.15 ± 2.44	3.49 ± 3.81	0.799	4.15 ± 2.44	6.01 ± 3.48	0.037*
5	4.63 ± 2.72	3.97 ± 4.09	0.646	4.63 ± 2.72	7.25 ± 4.21	0.037*
10	5.11 ± 2.62	3.39 ± 3.46	0.241	5.11 ± 2.62	6.44 ± 3.99	0.037*
15	4.89 ± 2.29	3.18 ± 3.13	0.333	4.89 ± 2.29	6.55 ± 3.89	0.037*
20	5.14 ± 2.09	3.63 ± 3.66	0.386	5.14 ± 2.09	7.07 ± 4.35	0.047*
25	5.31 ± 2.05	4.56 ± 3.53	0.333	5.31 ± 2.05	7.64 ± 4.23	0.037*
30	5.33 ± 2.43	4.18 ± 4.54	0.445	5.33 ± 2.43	7.82 ± 3.51	0.047*

Wilcoxon Signed Ranks Test.

Diff. = Right Mean IP–Left Mean IP.

*p < 0.05, significant difference between groups.

The unit of the asymmetries is mmHg.

### Temporal changes in IP characteristics

**Fig 2:**
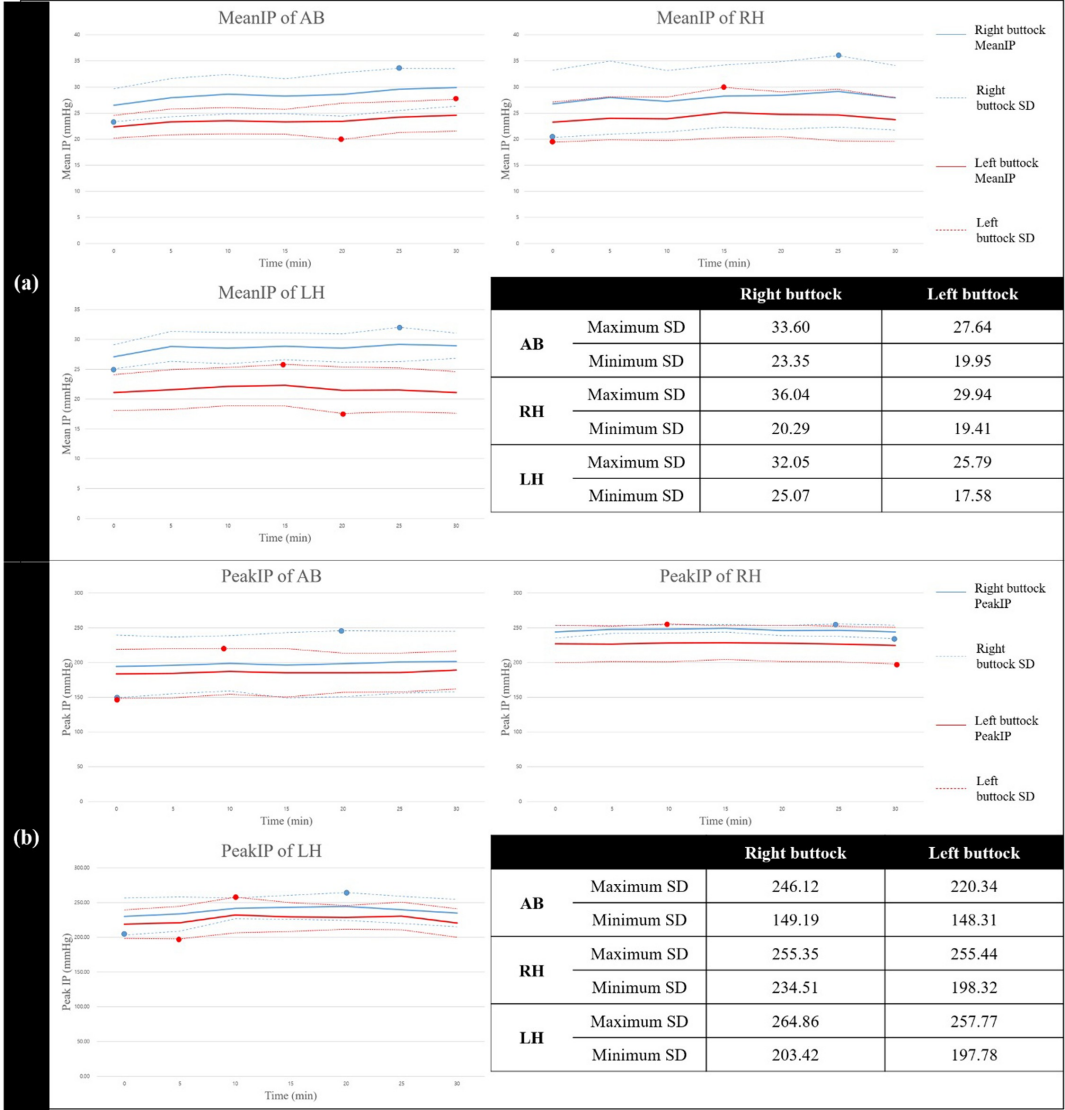
Temporal changes in Mean IPs (a) and Peak IPs (b). The solid blue lines represent the average (bold) of the IPs on the right buttock with the standard deviation (dashed line), and the solid red lines represent the average (bold) of the IPs with the standard deviation (dashed line).

The results of the related-samples Friedman’s two-way analysis of variance showed differences between the temporal changes in the IP characteristics on the right and left buttocks separately. For all groups, the mean IPs on both the buttocks show significant temporal changes (*p* < 0.05) (Table 5 and Fig 2A). The peak IPs on both the buttocks for all groups, as shown in Fig 2B, present insignificant temporal changes (*p* > 0.05) due to large variations of the peak IP values. The peak IP coordinates on both the buttocks for all groups also show insignificant temporal changes (*p* > 0.05).

**Table 5:** The related-samples Firedman’s two-way analysis of variance results for the mean IPs on both buttocks among the groups.

Type	Buttocks side	Analysis results			
		N	df	X2	p-value
AB	Right buttocks	10	6	29.54	0.000*
	Left buttocks	10	6	31.29	0.000*
RH	Right buttocks	12	6	20.86	0.002*
	Left buttocks	12	6	14.79	0.022*
LH	Right buttocks	13	6	33.23	0.000*
	Left buttocks	13	6	13.62	0.034*

N: sample size (the number of subjects AB, RH, LH).

Df: degree of freedom (7(0~30min)-1 = 6).

X2: Chi-square.

*p < 0.05, significant difference between groups.

## Discussion

In this study, for both hemiplegic groups, significant right-sided asymmetries were reported at all sitting periods for the mean IPs on the buttocks. Interestingly, significant right-sided asymmetry of the mean IP on the buttocks was observed even for AB. The right-sided asymmetry of the mean IP on the buttocks during sitting could be a natural characteristic of human posture based on anatomical research [14–17]. For example, Badii et al. [14] measured the distances from the right and left iliac crests to the acetabulum for 323 patients (150 females and 173 males) using CT scans. The frequency of right-sided pelvic obliquity was significantly higher than that of left-sided pelvic obliquity when people sat on a chair. Consequently, the intrinsic anatomical conditions can contribute to the observed right-sided asymmetries of the mean IP on the buttocks during sitting for the RH, LH, and AB.

This result indicates that the asymmetries of the mean IP on the AB’s buttocks cause right-sided weight relieving while sitting.

Despite constraining weight shifting, the buttocks of participants could have subtle movements. Although changes in the peak IP coordinates of the buttocks were not detected in this study, there would be movements of the peak IP coordinate in the normal (perpendicular) direction to the contact surface between the buttocks and the seat. A change in the peak IP value on a buttock is directly related to the normal displacement change of the contact surface at the peak IP coordinate. In general, the displacement change is a very small kinematic value caused by the buttock-tissue deformation. However, after conversion to the IP, which is a kinetic value (IP = a stiffness of the tissue per unit area × a normal displacement change at an IP coordinate), the normal displacement change can be detected as the peak IP change. Therefore, temporal changes in the peak IP could be useful to observe the subtle normal displacements of the contact surface at the peak IP coordinates. Therefore, the largest variation of the standard deviations (SDs) of the peak IP during the testing period (LVSD = the maximum SD—the minimum SD) can be used to observe subtle buttock movements.

For AB, as indicated in Fig 2B, the LVSD on the right buttock (97 mmHg) is higher than that on the left buttock (72 mmHg). Because AB has no neuromuscular disorders, the LVSD values for AB could be considered as reference values for both buttocks, representing usual movements for relieving the mean IP. The ratio of LVSD on the left buttock to that on the right buttock for the AB is 1.35. This result implies that the range of normal displacement of the contact surface at the peak IP coordinate on the right buttock is 1.35 times larger than that on the left buttock. Therefore, the ratio could represent the physiological pattern of the AB to relieve the right-sided asymmetric mean IP, and pathologies would cause a physiological pattern if a deviation from the ratio of AB exists.

For the RH, as indicated in Fig 2B, the LVSD on the right buttock, 22 mmHg, is lower than that on the left buttock, 57 mmHg. The LVSD values are substantially lower than those of the AB. Therefore, the normal displacements of the contact surfaces at the peak IP coordinates on both buttocks are minimal compared to those of the AB. Thus, the mean IPs for both buttocks of the RH could not be relieved compared to those of the AB. The ratio of LVSD on the left to the right buttock for the RH is 0.386. Moreover, the ratio of the RH is smaller than that of the AB. The paralytic neuromuscular conditions in the affected right buttock of the RH can be the reason for the narrow bandwidth of LVSD and the small value of the ratio. These would be caused by impairments of the left hemisphere, which inputs the sitting posture command to the musculoskeletal processes and the feedback for the sitting posture adjustments.

Particularly, the significantly small LVSD value on the right buttock of the RH indicates that the left hemisphere did not process the avoidance intention on the affected-side weight-bearing. Thus, the intrinsic right-sided asymmetry presented in AB results in the affected-side weight-bearing of the RH. Note that the RH has a stable sitting posture [18]. However, the stable sitting posture of the RH that bears the weight on the affected side buttock could cause ischemic conditions on the right buttock if sitting is prolonged.

For the LH, as indicated in Fig 2B, the LVSD of 61 mmHg and 60 mmHg on the right and left buttocks are similar to that of the AB’s left buttock. Moreover, the normal displacements of both buttocks were similar to that of the AB’s left buttock. However, the LH has disorders of the proprioceptive processes of the right hemisphere while having a sound sitting posture command and adjustment feedback of the left hemisphere. Errors between the sitting posture command and the feedback from the inaccurate proprioception result in an unintentional sitting posture of the LH. The resulting inadequate sitting postures are addressed by the feedback mechanism following the command. However, the errors are regenerated due to the disordered proprioception. Consequently, the alternating relatively large normal displacements on the buttocks occurred for the LH. An LVSD ratio of 1 indicates an alternating normal displacement behavior. Therefore, the LH showed apparent unstable sitting postures [18]. On the contrary, the apparent instability could help relieve the mean IPs for both the buttocks of LH. Because the left hemisphere is in charge of the avoidance intention on the affected-side weight-bearing of the LH, the LH shows the overly right-sided asymmetric mean IP increasing the intrinsic right-sided asymmetry.

As shown in Fig 2A, the mean IPs on both the buttocks for all groups show significant temporal changes. The mean IPs on both the buttocks for the AB gradually increase over time. In contrast, the mean IPs on both the buttocks for the RH and LH show sinusoidal increasing and decreasing fluctuation with respect to time. Therefore, the temporal mean IPs on the buttocks of persons with hemiplegia were different from those on AB. Moreover, the RH and LH presented significantly higher peak IPs on both buttocks than the AB for all sitting periods. However, the peak IPs on both buttocks for the RH were the same as those for the LH. The peak IPs on the unaffected-side buttocks of RH and LH were significantly higher than those on the buttocks of AB. From the results for the temporal behavior of the mean IP and higher peak IP of the RH and LH, the unaffected-side buttock of RH and LH could be affected by the pathologies of the affected side.

The experiments revealed that the apparent stability of the sitting posture for the RH could not relieve the mean IP on both buttocks. Particularly, the weight of RH started in the affected-side right buttock. In addition, adjustments and avoidance of the intention of the sitting posture cannot be initiated and processed by the RH. Furthermore, the RH showed significantly higher peak IPs for both the buttocks than those of the AB.

On the contrary, the instability of the sitting posture of the LH could relieve the mean IP on both buttocks. However, the overly right-sided asymmetric mean IP on the unaffected buttock must be redistributed to the left buttock for a balanced sitting posture. Thus, the significantly high peak IP on both buttocks compared to those of the AB should be lowered. Moreover, a buttock positioning orthosis is required to balance the buttocks for persons with LH, for instance, a passive seat orthosis that does not use the floatation principle is recommended for the LH to stabilize the sitting posture and reduce the peak IP on both buttocks.

In this study, only hospitalized male hemiplegic patients who were in the acute and recovery stages (maximum of eight weeks post-stroke), as indicated in the methods, were evaluated. The recruited hemiplegic patients in the acute and recovery stages essentially used wheelchairs while undergoing functional recovery training, such as walking and sitting. The extensive uses of wheelchairs for mobility by hemiplegic patients in the acute and recovery stage have been indicated in previous studies [19, 20]. Since prolonged sitting time is unavoidable for stroke patients in the acute and recovery stages, we chose hemiplegic patients in these stages for this study. In addition, since the total number of patients who met the conditions was limited, a non-parametric test was performed after the normality test. As the result of Shapiro-wilk test for effective sample size and normality test was p <0.05, it did not satisfy the normal distribution, so the analysis was conducted with a non-parametric test corresponding to the parametric test. This small sample size could be the limitation of this study.

Biomechanically, the results obtained in this study are not representative of the IP characteristics of females as the buttocks anatomy of females and males differs. Thus, research is recommended to understand the buttock IP characteristics of female patients with hemiplegia. The experiments were performed for only 30 min, which is a relatively short period for determining the full effects of temporal posture variations in IP characteristics. Nevertheless, rehabilitation doctors recommended the period used for the safety of patients. Longer-duration sitting experiments are recommended for future studies as long as the safety of persons with hemiplegic is guaranteed.

## Conclusion

Recently, several researchers have reported that the affected side of hemiplegic patients affects the unaffected side [21, 22]. However, there was no study on the difference between left and right hemiplegia. In this study, it was assumed that LH and RH would have different weight-releasing tendencies during sitting owing to different neuromusculoskeletal conditions. Thus, this study investigated the temporal characteristics of peak interface pressure (IP) on right and left buttocks to understand weight-releasing tendencies for RH, LH and AB sitting in a wheelchair for 30 min.

In the previous studies, the asymmetry of the pelvis due to the biased posture control causes the lowering of the ability to balance the sitting posture. In addition, 85% of stroke patients developed hemiplegia, and the ability to weight-shifting on the affected side was reduced, making it difficult to control postural balance [23, 24]. Boyle, Colin J., et al notes that pressure equalization is a more effective way to reduce the prevalence of pressure ulcers [25].

Therefore, separate and specific rehabilitation for RH and LH is required to control the IP on the buttocks.

Based on the results of this study, in the case of RH, an approach through pressure equalization of the left buttock showing excessive peak IP difference is required to reduce the prevalence of pressure ulcers. Right buttock of RH needs periodic posture changes to avoid prolonged skin contact. Conversely, LH needs to reduce friction and wetting due to prolonged skin contact by giving periodic pressure changes or posture changes in both buttocks.

## Abbreviations

IP: Interface pressure
RH: Right hemiplegia
LH: Left hemiplegia
AB: Able-bodied individuals
SD: Standard deviation
